# The effects of phenolic glycosides from *Betula platyphylla* var. *japonica* on adipocyte differentiation and mature adipocyte metabolism

**DOI:** 10.1080/14756366.2018.1491846

**Published:** 2018-08-21

**Authors:** Joo Young Huh, Seulah Lee, Eun-Bi Ma, Hee Jeong Eom, Jiwon Baek, Yoon-Joo Ko, Ki Hyun Kim

**Affiliations:** a College of Pharmacy, Chonnam National University, Gwangju, Republic of Korea;; b School of Pharmacy, Sungkyunkwan University, Suwon, Republic of Korea;; c Laboratory of Nucear Magnetic Resonance, National Center for Inter-University Research Facilities (NCIRF), Seoul National University, Gwanak-gu, Seoul, Republic of Korea

**Keywords:** *Betula platyphylla* var. *japonica*, catechin glycoside, phenolic glycoside, adipocyte differentiation, mature adipocyte metabolism

## Abstract

*Betula platyphylla* var. *japonica* (Betulaceae) has been used traditionally in Asian countries for the treatment of inflammatory diseases. A recent study has reported a phenolic compound, platyphylloside from *B. platyphylla*, that shows inhibition on adipocyte differentiation and induces lipolysis in 3T3-L1 cells. Based on this finding, we conducted phytochemical analysis of the EtOH extract of the bark of *B. platyphylla* var. *japonica,* which resulted in the isolation of phenolic glycosides (**1**–**4**). Treatment of the isolated compounds (**1**–**4**) during adipocyte differentiation of 3T3-L1 mouse adipocytes resulted in dose-dependent inhibition of adipogenesis. In mature adipocytes, arylbutanoid glycosides (**2**–**4**) induced lipolysis related genes HSL and ATGL, whereas catechin glycoside (**1**) had no effect. Additionally, arylbutanoid glycosides (**2**–**4**) also induced GLUT4 and adiponectin mRNA expression, indicating improvement in insulin signaling. This suggests that the isolates from *B. platyphylla* var. *japonica* exert benefial effects in regulation of adipocyte differentiation as well as adipocyte metabolism.

## Graphical Abstract

**Figure:**
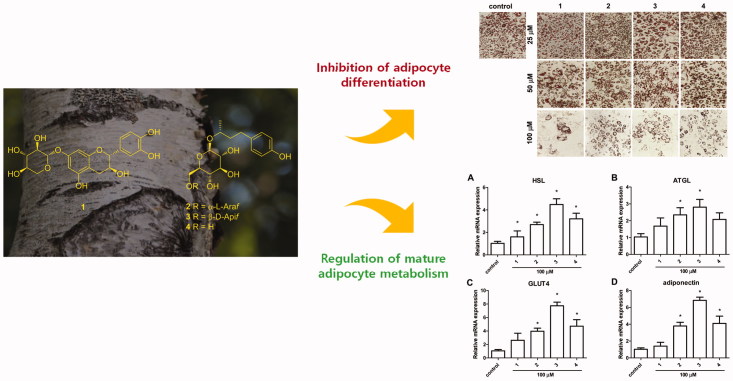

## Introduction

Obesity is one of the most prevalent chronic diseases worldwide^1^. It is a state of excessive fat accumulation in the adipose tissue which results in metabolic diseases such as insulin resistance, type 2 diabetes mellitus, and cardiovascular diseases^2^. Development of obesity involves two routes, increase in adipocyte size (hypertrophy) and increase in adipocyte number (hyperplasia)^3^, and both are considered as targets for the treatment of obesity. Increase in adipocyte number is achieved through adipocyte differentiation. The adipogenic process involves a variety of transcriptional factors, such as peroxisome proliferator-activated receptor (PPARγ), CCAAT/enhancer binding proteins (such as C/EBPα), and fatty acid binding protein 4 (FABP4), and these factors in concert induce intracellular storage of triglycerides^4^. Mature adipocytes are primarily involved in the regulation of energy homeostasis by buffering lipid metabolites and secreting adipokines such as leptin and adiponectin^5^. Of note, adiponectin is a well-known anti-inflammatory adipokine, which regulates not only local adipocyte insulin sensitivity but also the whole body metabolism through enhancing energy expenditure on peripheral tissues, such as liver and muscle^5^. Therefore, dysregulation of adipokines along with inflammation in adipose tissue ultimately leads to the development of metabolic diseases such as insulin resistance^6^.

*Betula platyphylla* var. *japonica* (Miquel) Hara (Betulaceae), which is also known as Asian white birch, is distributed throughout Asian countries including Korea, Japan, China, and eastern Siberia^7–9^. The bark of *B. platyphylla* var. *japonica* has been used as a traditional medicine in these countries, for the treatment of a wide variety of inflammatory diseases, including pneumonia, choloplania, nephritis, chronic bronchitis, arthritis, and dermatitis^7–10^, as well as for the relief of heat and cough^11^. In previous studies, the extract of *B. platyphylla* var. *japonica* is reported to have diverse therapeutic effects including neuroprotective^12^, antioxidant^10^, anticancer^13^, and hepatoprotective activities^10^. In our continuing endeavor to discover bioactive compounds from natural resources^14–18^, we have taken our interest in identifying bioactive compounds from the bark of *B. platyphylla* var. *japonica*, accordingly on the above findings. Phytochemical studies of the bark of *B. platyphylla* var. *japonica* have revealed the presence of triterpenoids^19–21^ and phenolic compounds^11^. Particularly, triterpenoids have been known to be the major bioactive components of the bark of *B. platyphylla* var. *japonica*, where their biological investigations had been limited mostly to anticancer activity^19–21^. As the main components of the bark of *B. platyphylla* var. *japonica*, triterpenoids were also identified from the source in our recent studies^8^ ^,^ ^9^, where the chemical characterization of antioxidant triterpenoids combined with phenylpropanoid unit as well as cytotoxic triterpenoids against several human tumor cells were reported^8^ ^,^ ^9^. Despite repetitive reports of the identification of triterpenoids from *B. platyphylla* var. *japonica*, the chemical investigation of other types of components such as flavonoids and/or phenolics from *B. platyphylla* var. *japonica* are relatively uninvestigated. Meanwhile, a recent paper reported a phenolic compound, platyphylloside from *B. platyphylla*, which is a diarylheptanoid that shows inhibitory effects on adipocyte differentiation and induces lipolysis in 3T3-L1 cells, suggesting its potential application in not only the prevention but also in the treatment of obesity^22^. This have led to our increasing interest of phenolic compounds in the bark of *B. platyphylla* var. *japonica*, in hoping for the expansion of applications of the *B. platyphylla* var. *japonica* bark as an anti-obesity agent.

**Figure 1.**
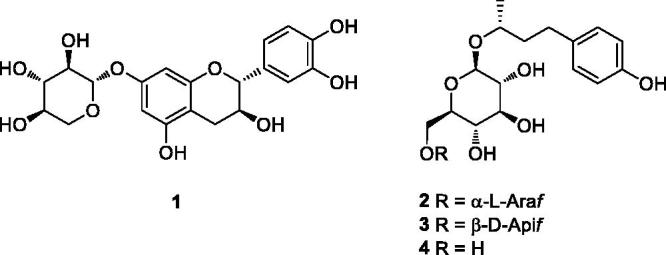
Chemical structures of compounds **1–4** identified from the bark of *B. platyphylla* var. *japonica*. Araf: arabinofuranosyl; Apif: apiofuranosyl.

In this study, phytochemical analysis of the EtOH extract of bark of *B. platyphylla* var. *japonica* led to the isolation of phenolic glycosides including a catechin glycoside (**1**) and three arylbutanoid glycosides (**2**–**4**), structures of which were elucidated based on the NMR spectroscopic data and LC/MS analysis (Figure 1). In addition, the effects of phenolic glycosides identified from the bark of *B. platyphylla* var. *japonica* on adipocyte differentiation and mature adipocyte metabolism were investigated. The current study reports the isolation and structural elucidation of phenolic glycosides and the evaluation of regulating effects of the isolates on adipocyte differentiation and mature adipocyte metabolism.

## Materials and methods

### General experimental procedures

Optical rotations were measured using a Jasco P-1020 polarimeter (Jasco, Easton, MD). IR spectra were recorded with a Bruker IFS-66/S FT-IR spectrometer (Bruker, Karlsruhe, Germany). UV spectra were acquired on an Agilent 8453 UV-visible spectrophotometer (Agilent Technologies, Santa Clara, CA). Nuclear magnetic resonance (NMR) spectra were recorded with a Bruker AVANCE III 700 NMR spectrometer operating at 700 MHz (^1^H) and 175 MHz (^13^C) (Bruker, Karlsruhe, Germany). Semi-preparative HPLC was performed using a Shimadzu Prominence HPLC System with SPD-20A/20AV Series Prominence HPLC UV-Vis detectors (Shimadzu, Tokyo, Japan) with a flow rate of 2 ml/min. LC/MS analysis was performed on an Agilent 1200 Series HPLC system equipped with a diode array detector and 6130 Series ESI mass spectrometer using an analytical Kinetex C18 100 Å column (100 × 2.1 mm i.d., 5 µm; Phenomenex, Torrance, CA). Silica gel 60 (70-230 mesh and 230–400 mesh; Merck, Darmstadt, Germany) and RP-C_18_ silica gel (Merck, 40-63 µm) were used for column chromatography. Merck precoated silica gel F_254_ plates and RP-18 F_254s_ plates were used for TLC. Spots were detected after TLC under UV light or by heating after spraying with anisaldehyde-sulfuric acid.

### Plant material

The bark of *B. platyphylla* var. *japonica* were collected from Danyang, Chungcheongbuk-do, Korea, in October 2014. The material was identified by one of the authors (K. H. Kim). A voucher specimen (NM-14-063) was deposited in the herbarium of the Natural Medicine Research Center of Richwood Pharmaceutical Company, Ltd., Seoul, Korea.

### Extraction and isolation

The air-dried bark of *B. platyphylla* var. *japonica* (200 g) was chopped and extracted with 80% EtOH at room temperature three times each for 24 h. The extract was filtered and the filtrate was evaporated under reduced pressure using a rotavapor to obtain the crude extract (17 g), which was then successively solvent-partitioned with CHCl_3_, EtOAc, and *n*-BuOH, yielding 12.5, 1.3, and 1.5 g of residue, respectively. A small aliquots of three solvent-partitioned fractions were sequentially injected into LC/MS eluted with a gradient solvent system of MeOH/H_2_O (1:9 – 1:0, flow rate of 0.3 ml/min, UV 210 nm), which revealed the presence of several peaks for phenolic compounds from *n*-BuOH-soluble fraction as main components by comparison with our house-built UV library in LC/MS. Accordingly, the *n*-BuOH-soluble fraction (1.5 g) was subjected to silica gel column chromatography using a gradient solvent system of CH_2_Cl_2_/MeOH/H_2_O (20:4.5:0.5–30:10:1–10:5:1) to yield ten fractions (A1–A10). Fraction A5 (620 mg) was further fractionated using RP-C18 silica gel column chromatography with a gradient solvent system of MeOH/H_2_O (4:6–1:0), which gave five subfractions (A51–A55). Subfraction A52 (140 mg) was then purified by the semi-preparative HPLC purification (2 ml/min, 50% MeOH) using a Phenomenex Luna C18 column (250 × 10 mm i.d., 10 µm) to yield compound **4** (40 mg). Fraction A7 (380 mg) was subjected to RP-C18 silica gel column chromatography eluting with a gradient solvent system of MeOH/H_2_O (4:6–1:0), to give five subfractions (A71–A75). Subfraction A72 (130 mg) was further purified by the means of semi-preparative HPLC (2 ml/min, 40% MeOH) using a Phenomenex Luna C18 column (250 × 10 mm i.d., 10 µm), which yielded compounds **2** (18 mg) and **3** (30 mg). Fraction A8 (450 mg) was fractionated on RP-C18 silica gel column using a gradient solvent system of MeOH/H_2_O (4:6–1:0), yielding six subfractions (A81–A86). Subfraction A82 (55 mg) was purified by semi-preparative HPLC purification (2 ml/min, CH_2_Cl_2_/MeOH/H_2_O, 20:4.5:0.5) equipped with Apollo silica column (250 × 10 mm i.d., 5 µm; Apollo, Manchester, UK) to afford compound **1** (18 mg). All isolated compounds were identified by comparison with previously reported NMR spectroscopic data and LC/MS analysis.

### Quantitative analysis of the isolated compounds by LC/MS

The detection of each isolated compound (**1**–**4**) was analyzed using LC/MS, Agilent 1200 Series analytical system (Agilent Technologies, Santa Clara, CA) equipped with a photodiode array (PDA) detector combined with a 6130 Series ESI mass spectrometer. The EtOH extract (1.0 mg) of *B. platyphylla* var. *japonica* was dissolved in 1 ml MeOH to provide a solution of 1000 µg/mL. The solution was filtered through a 0.45 mm hydrophobic PTFE filter and finally analyzed by LC/MS using a Kinetex C18 column (2.1 × 100 mm, 5 µm; Phenomenex, Torrance, CA) set at 25 °C. The mobile phase consisting of formic acid in H_2_O [0.1% (v/v)] (A) and methanol (B) was delivered at a flow rate of 0.3 ml/min by applying the following programmed gradient elution: 10%-90% (B) for 30 min, 100% (B) for 1 min, 100% (B) isocratic for 10 min, and then 10% (B) isocratic for 10 min, to perform post-run reconditioning of the column. Calibration curves and linear regression equations were generated for each compound (**1**–**4**) as external standards. Quantification of each compound was based on the peak area obtained from the MS detection and calculated as equivalents of the standard. All contents are expressed as grams per 100 g of extract weight.

### Cell culture and differentiation

The 3T3-L1 cell line was purchased from the American Type Culture Collection (Rockville, MD). Cells were cultured in Dulbecco's modified Eagle's medium (DMEM; Hyclone, Logan, UT) supplemented with 10% fetal bovine serum (FBS; Hyclone) and 1% penicillin/streptomycin and incubated at 37 °C in 5% CO_2_. 3T3-L1 cells were differentiated in a standard protocol where differentiation media 1, 2, and 3 were used^23^. Briefly, two days after the 3T3-L1 preadipocytes reached 100% confluency (day 0), cells were treated with differentiation media 1 where 5 µg/mL insulin (Sigma, St. Louis, MO), 0.25 mM dexamethasone (Sigma), and 0.5 mM 1-methyl-3-isobutylxanthine (Sigma) was added to 10% FBS DMEM media. After 2 days (day 2), cells were changed to differentiation media 2 which consisted of 5 µg/mL insulin added to 10% FBS DMEM. Thereafter, medium was replaced every 48 h with fresh 10% FBS DMEM media (differentiation media 3) until day 8. Cells were treated with isolated compounds from on day 0 and day 2 at the same time the cells were changed to differentiation media 1 and 2 (for 4 days in total) to examine their effects on adipogenesis. For determination of the effects on differentiation markers expression, the cells were treated with 50 and 100 µM of isolated compounds. For determination of the effects on cell differentiation through Oil Red O staining, the cells were treated with 25, 50, and 100 µM of isolated compounds. The isolated compounds were dissolved in DMSO, and therefore the same amount of DMSO was treated to the control group.

### Cell viability

Cell viability was determined using the 3-(4,5-dimethylthiazol-2-yl)-2,5-diphenyl-tetrazolium-bromide (MTT) assay^15,24–26^. 3T3-L1 preadipocytes were treated with various concentration of isolated compounds for 24 h. Then MTT reagent was added to a final concentration of 0.5 mg/mL for two hours. Then DMSO was added for 10 min before measuring the absorbance at 540 nm.

### Oil red O (ORO) staining

Eight days after induction of differentiation (day 8), the cells were fixed with 10% formalin for 1 h. Then the cells were stained with 0.3% Oil Red O solution (Sigma) for 1 h. ORO was harvested with 100% isopropanol for quantification.

### Western blotting

Cells at day 4 were harvested and lysed in RIPA buffer (Thermo Scientific, Rockford, USA) and separated by SDS-PAGE and transferred to PVDF membrane. The membranes were incubated with primary antibodies (Cell Signaling, Danvers, USA; FABP4, PPARγ, C/EBPα) overnight. The blots were detected using LAS-3000 (Fuji photo film, Tokyo, Japan).

### Gene expression analysis

Cells at day 8 were treated with 100 µM of isolated compounds for 24 h. Total RNA was extracted from cells using TRI Reagent (MRC, TR118, Cincinnati, OH). cDNA was synthesized using TOPscript™ RT DryMIX (Enzynomics, Daejeon, Korea). mRNA levels were measured by real-time PCR using Rotor-Gene Q (QIAGEN) with 20 µL reaction volume consisting of cDNA transcripts, primer pairs, and TOPreal SYBR Green PCR Kit (Enzynomics, Daejeon, Korea). The gene expressions were normalized to 18S rRNA levels.

### Statistical analysis

All statistical analysis was performed using Statview software. Mean values obtained from each group were compared by ANOVA. *p* values of <0.05 was used as the criterion for a statistically significant difference.

## Results and discussion

### Isolation and structure characterization of phenolic compounds

A small aliquots of CHCl_3_, EtOAc, and *n*-BuOH-soluble fractions obtained by solvent-partitioning of the EtOH extract were examined in LC/MS analysis for phenolics as desired compounds, and the LC/MS analysis of the fractions revealed the presence of several peaks for phenolic compounds from *n*-BuOH-soluble fraction as main components by comparison with our house-built UV library in LC/MS. Accordingly, we conducted phytochemical investigation of the *n*-BuOH-soluble fraction to isolate phenolic compounds, which was achieved by repeated column chromatography over RP-C18 silica gel and HPLC purification experiment. The phytochemical analysis led to the isolation of phenolic glycosides including a catechin glycoside (**1**) and three arylbutanoid glycosides (**2**–**4**) (Figure 1). The isolated compounds were identified as (+)-catechin 7-*O*-*β*-D-xylopyranoside (**1**)^27^, (2*R*)-4-(4-hydroxyphenyl)-2-butanol 2-*O*-*α*-L-arabinofuranosyl-(1 → 6)-*β*-D-glucopyranoside (**2**)^28^, (2*R*)-4-(4-hydroxyphenyl)-2-butanol 2-*O*-*β*-D-apiofuranosyl-(1 → 6)-*β*-D-glucopyranoside (**3**)^29^, and (1*R*)-3-(4-hydroxyphenyl)-1-methylpropyl *β*-D-glucopyranoside (**4**)^30^ by comparison with previously reported NMR spectroscopic data and LC/MS analysis (Figure 1). Quantitative analysis of the isolated compounds (**1**–**4**) by LC/MS exhibited contents of compounds **1**–**4** with values of 3.99 ± 0.31, 9.58 ± 0.09, 1.18 ± 0.02, and 0.97 ± 0.09 g/100 g of the EtOH extract weight, respectively (Table 1). According to our quantitative analysis, compound **2** was the most predominant component in the bark of *B. platyphylla* var. *japonica*, and the other compounds also accounted for relatively high amount of the plant source (see Supplemental data).

**Table 1.** Contents (g/100 g of EtOH extract weight) of the isolated compounds (**1**–**4**) in the EtOH extract of *B. platyphylla* var. *japonica*.^a^

Sample	Content
1	3.99 ± 0.31
2	9.58 ± 0.09
3	1.18 ± 0.02
4	0.97 ± 0.09

^a^Each result shown in the table is the mean of three replicated measurements.

In previous studies based on the biological activities of the isolated compounds, (+)-catechin 7-*O*-*β*-D-xylopyranoside (**1**) was found to reduce AGEs-BSA cross-linking to collagen where this cross-linking plays an important role in the arterial and myocardial stiffening, which eventually increases cardiac risks related with aging and diabetes^31^. An arylbutanoid glycoside, (2*R*)-4-(4-hydroxyphenyl)-2-butanol 2-*O*-*β*-D-apiofuranosyl-(1 → 6)-*β*-D-glucopyranoside (**3**), was reported to increase the function of osteoblastic MC3T3-E1 cells, suggesting its possible application for the prevention and treatment of osteoporosis^32^, and also showed remarkable inhibitory activity against the degranulation of RBL-2H3 by antigen stimulation, which can potentially be used for the treatment of IgE–FcεRI interaction related allergic disorders^33^. Another arylbutanoid glycoside, (1*R*)-3-(4-hydroxyphenyl)-1-methylpropyl *β*-D-glucopyranoside (**4**), was reported to exhibit strong antioxidative activities against DPPH radical-scavenging and reducing power (FRAP)^34^, as well as to ameliorate skin inflammation through inhibition of NF-κB, MAPK, and PI3K/Akt signaling inTNF-α/IFN-γ-stimulated keratinocytes^35^. However, the regulating effects of the isolated compounds on adipocyte differentiation and mature adipocyte metabolism have not been reported to date.

### The effect of isolated phenolic compounds on adipocyte differentiation

**Figure 2.**
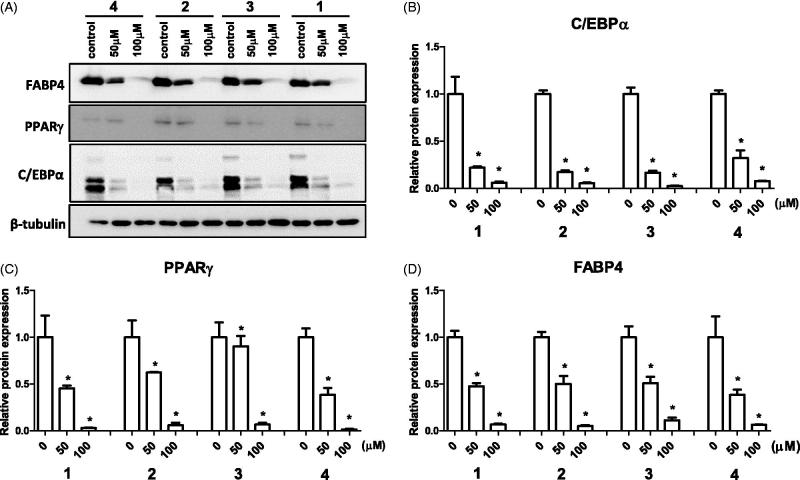
Effect of isolated compounds from the bark of *B. platyphylla* var. *japonica* on markers of adipocyte differentiation. 3T3-L1 preadipocytes were treated with various concentrations of compounds **1–4** from start of adipocyte differentiation (day 0) until fourth day. Representative image (A) and quantification (B–D) of adipogenic markers C/EBPα, PPARγ, and FABP4 protein expression on day 4 of differentiation. Values are means ± SE of three experiments. *p < 0.05 versus control (no treatment).

**Figure 3.**
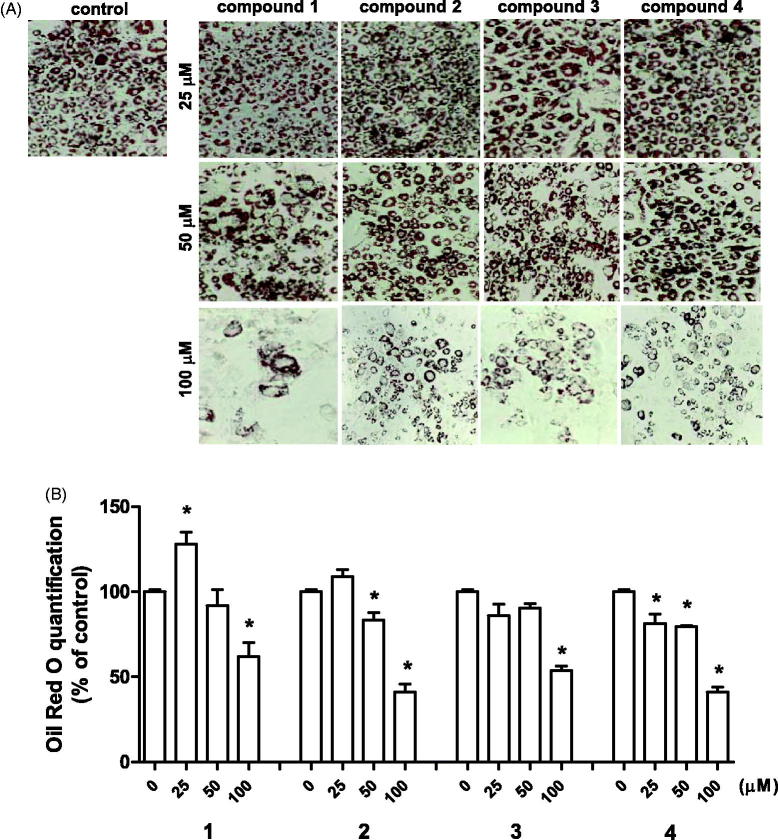
Effect of isolated compounds from the bark of *B. platyphylla* var. *japonica* on lipid accumulation during adipocyte differentiation. 3T3-L1 preadipocytes were treated with various concentrations of compounds **1–4** from start of adipocyte differentiation (day 0) until fourth day. (A) The representative Oil Red O staining pictures of differentiated adipocytes on day 8. (B) Quantification of Oil Red O staining. Values are means ± SE of 3 experiments. *p < 0.05 versus control (no treatment).

First to check whether the isolated compounds had any effect on cell viability, 3T3-L1 preadipocytes were treated with various doses of the compounds (12.5–200 µM) for 24 h. Since no cellular toxicity is observed up to 200 µM for all compounds (data not shown), we have selected 50 and 100 µM dose for the subsequent experiments. To examine the effect of phenolic compounds on adipogenesis, the compounds were treated to 3T3-L1 cells at the start of differentiation process until fourth day, which is the critical period for adipogenic determination and differentiation. The protein levels of adipogenic markers, FABP4, PPARγ, and C/EBPα were measured at day 4^4^. The result shows that all four phenolic compounds downregulated these markers FABP4, PPARγ, and C/EBPα levels in a dose-dependent manner, where at 100 µM the markers were fully suppressed (Figures 2(A–D)). The Oil Red O staining at day 8 revealed that the isolated compounds inhibited accumulation of intracellular lipids (Figure 3(A)). Also, the percentage of differentiated cells were lower at higher concentration of phenolic compounds. The quantification of Oil Red O stain confirmed the dose-dependent inhibitory effect of the isolated compounds on 3T3-L1 cells (Figure 3(B)). The degree of inhibition did not vary among different compounds. In addition, at the concentration of 100 µM, the inhibitory effect was 40–50% compared to control (fully differentiated cells). The data confirmed that at high concentration of isolated phenolic compounds, the adipogenic process is inhibited through regulation of transcriptional factors.

### The effect of isolated phenolic compounds on mature adipocyte metabolism

**Figure 4.**
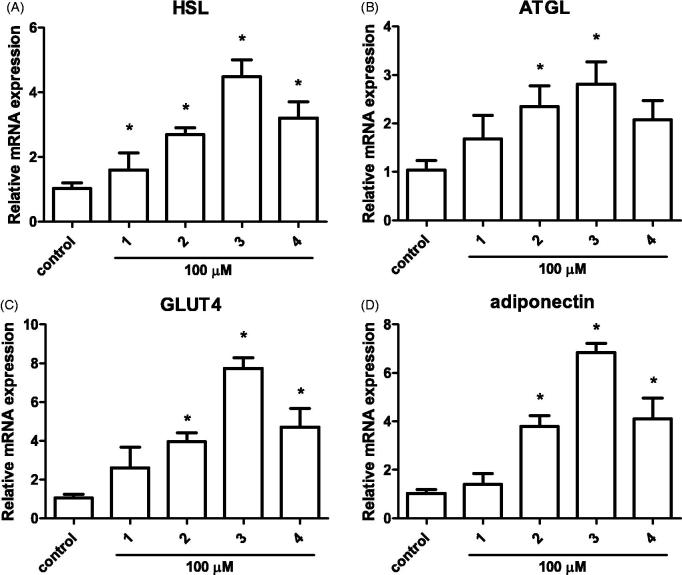
Effect of isolated compounds from the bark of *B. platyphylla* var. *japonica* on mature adipocyte gene expression. 3T3-L1 mature adipocytes were treated with 100 μM of compounds **1–4**. Gene expression was measured after 24 h incubation using real-time PCR. Values are means ± SE of three experiments. *p < 0.05 versus control (no treatment).

Previous studies have shown that adipocytes are sensitive to insulin and dysregulated adipocyte metabolism leads to adipocyte and whole body insulin resistance^6^. Along with the effect of phenolic compounds on adipocyte differentiation, the effects on mature adipocyte metabolism was examined. Fully differentiated adipocytes were treated with 100 µM of the isolated compounds for 24 h, and gene expression of lipolysis enzymes adipose triglyceride lipase (ATGL) and hormone-sensitive lipase (HSL) was measured. The hydrolysis of triglycerides in adipocytes is catalyzed by a cascade of lipolytic enzymes and ATGL and HSL constitute the first and second step in this cascade^36^. Treatment of arylbutanoid glycosides (**2**–**4**) significantly induced HSL mRNA expression where as catechin glycoside (**1**) had no effect (Figure 4(A)). Similarly, arylbutanoid glycosides (**2** and **3**) significantly induced ATGL mRNA expression whereas another arylbutanoid glycoside **4** had a trend for increase and catechin glycoside (**1**) had no effect (Figure 4(B)). These results imply that the arylbutanoid glycosides isolated from *B. platyphylla* var. *japonica* effectively induce hydrolysis of triglyceride which would be beneficial in losing weight by burning fat in adipocytes.

Obesity is considered detrimental since it causes metabolic disturbances in adipocytes as well as at the whole body level^2^. Therefore, the amelioration of insulin sensitivity in obese individual is a critical factor in treatment of metabolic syndrome. Numerous reports known to date has explored the pharmacological effects of the extracts from *Betula* species, and several components have shown anti-inflammatory and anti-oxidative effects^37^, which could potentially contribute to the beneficial role of *Betula* species in treatment of metabolic diseases. However, there are limited evidence on the direct role of the components from *B. platyphylla* on metabolic diseases such as obesity and diabetes. In a recent paper where platyphylloside from *B. platyphylla* was tested in 3T3-L1 adipocytes, similar effects to ours were observed where adipocyte differentiation markers PPARγ and C/EBPα were downregulated and lipolysis markers HSL and perilipin were also downregulated in mature adipocytes^23^. However, they have not examined whether markers of insulin sensitivity were affected in mature adipocytes. In our study, to examine whether the isolated phenolic compounds exert an anti-diabetic effect on mature adipocytes, we measured the gene expression levels of glucose transporter type 4 (GLUT4) and adiponectin. In line with the HSL and ATGL expression, GLUT4 and adiponectin mRNA levels were only induced by arylbutanoid glycosides (**2–4**) but not by catechin glycoside (**1**) (Figure 4(C,D)). Among isolated arylbutanoid glycosides, compound **3** had the largest fold change of expression. Adiponectin is an adipokine that decreases inflammation and oxidative stress in adipocytes and induce insulin sensitization in adipocytes and other peripheral tissues^38^. It is known that adiponectin exerts its biological effects through AMPK which regulates various genes related to insulin signaling, including GLUT4^4^. The fact that the isolated arylbutanoid glycosides upregulate GLUT4 and adiponectin expression imply that these compounds may exert anti-obesity and anti-diabetic effects through improvement in insulin signaling. Whether or not the change in expression levels lead to sensitization of insulin needs to be further studied in animal studies.

## Conclusions

In the current study, phytochemical analysis of the EtOH extract of bark of *B. platyphylla* var. *japonica* were carried out, which led to the isolation of phenolic glycosides including a catechin glycoside (**1**) and three arylbutanoid glycosides (**2**–**4**), structures of which were elucidated based on the NMR spectroscopic data and LC/MS analysis. Treatment of arylbutanoid glycosides (**2**–**4**) to mouse adipocytes resulted in inhibition of adipocyte differentiation and regulation of metabolic genes in mature adipocytes. These results suggest that the arylbutanoid glycosides, (2*R*)-4-(4-hydroxyphenyl)-2-butanol 2-*O*-*α*-L-arabinofuranosyl-(1 → 6)-*β*-D-glucopyranoside (**2**), (1*R*)-3-(4-hydroxyphenyl)-1-methylpropyl 6-*O*-D-apio-*β*-D-furanosyl-*β*-D-glucopyranoside (**3**), and (1*R*)-3-(4-hydroxyphenyl)-1-methylpropyl *β*-D-glucopyranoside (**4**) isolated from *B. platyphylla* var. *japonica* exerts benefial effects in regulation of adipocyte differentiation and adipocyte metabolism.

## Supplementary Material

Supplemental Material
