# Supplementary material for: The effects of phenolic glycosides from Betula platyphylla var. japonica on adipocyte differentiation and mature adipocyte metabolism
Source: J Enzyme Inhib Med Chem. 2018 Aug 21;33(1):1167–73. doi: 10.1080/14756366.2018.1491846 (PMC6104606; doi:10.1080/14756366.2018.1491846)
Supplement: Supplemental Material [file IENZ_A_1491846_SM1305.pdf]

Supplemental material

The effects of phenolic glycosides from *Betula platyphylla* var. *japonica* on adipocyte differentiation and mature adipocyte metabolism

Joo Young Huh[a,1], Seulah Lee[b,1], Eun-Bi Ma[a], Hee Jeong Eom[b], Jiwon Baek[b], Yoon-Joo Ko[c], Ki Hyun Kim[b,*]

[a]*College of Pharmacy, Chonnam National University, Gwangju 61186, Republic of Korea*

[b]*School of Pharmacy, Sungkyunkwan University, Suwon 16419, Republic of Korea*

[c]*Laboratory of Nucear Magnetic Resonance, National Center for Inter-University Research Facilities (NCIRF), Seoul National University, Gwanak-gu, Seoul 08826, Republic of Korea*

[1]These authors contributed equally to this work.

* Corresponding author: Ki Hyun Kim, Tel: +82-31-290-7700; Fax: +82-31-290-7730; E-mail: khkim83@skku.edu

LC/MS analysis of compounds 1-4

Stock solutions of compounds **1-4** were prepared by dissolving 1 mg of each compound in 1 mL methanol. Each solution was further diluted with methanol to provide a solution of 100 µg/mL. The solutions were filtered through a 0.45 mm hydrophobic PTFE filter and analyzed by LC/MS (Agilent Technologies, Santa Clara, CA, USA) using a LC-MS Agilent 1200 Series analytical system equipped with a photodiode array (PDA) detector combined with a 6130 Series ESI mass spectrometer. Analysis was performed by the injection of 15 µL of the sample using a Kinetex C18 column (2.1 × 100 mm, 5 µm; Phenomenex, Torrance, CA, USA) set at 25°C. The mobile phase consisting of formic acid in H_2O [0.1% (v/v)] (A) and methanol (B) was delivered at a flow rate of 0.3 mL/min by applying the following programmed gradient elution: 10%-90% (B) for 30 min, 100% (B) for 1 min, 100% (B) isocratic for 10 min, and then 10% (B) isocratic for 10 min, to perform post-run reconditioning of the column.

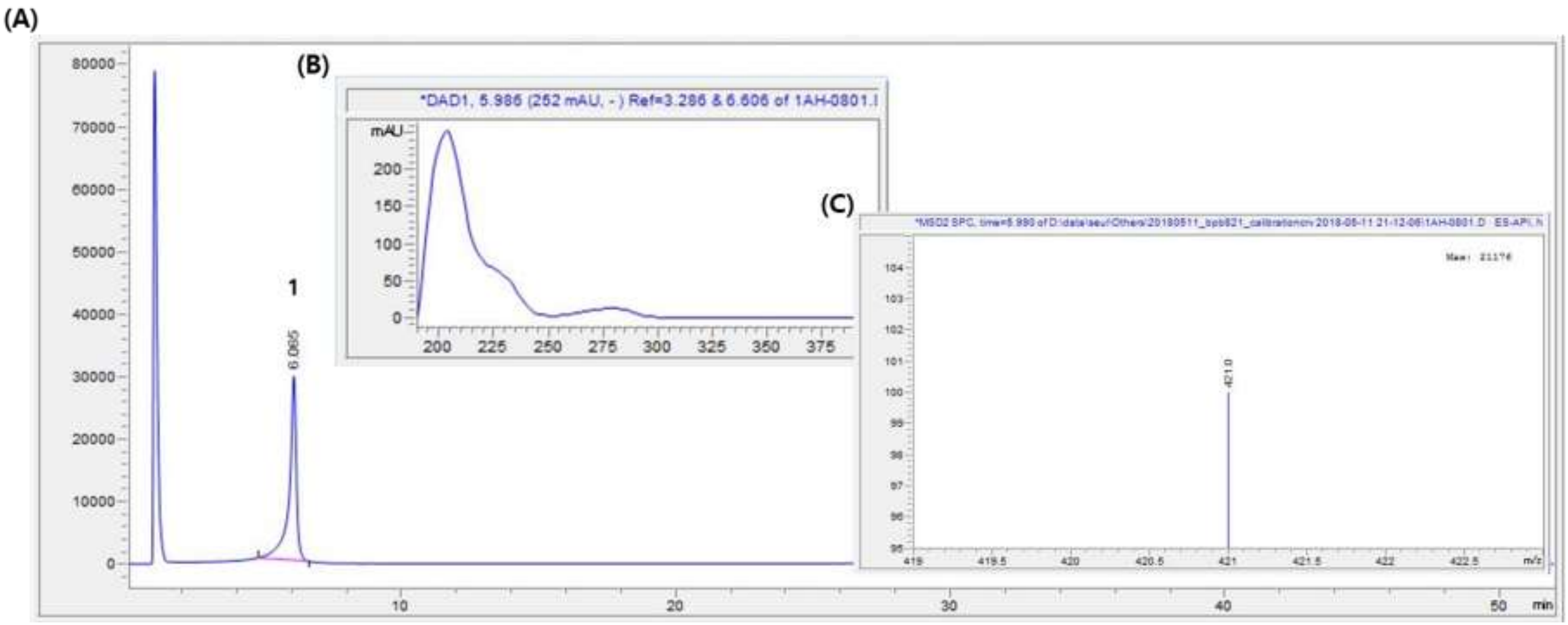

Figure S1. The LC/MS analysis of compound **1**. (A) UV chromatogram of LC/MS (detection wavelength was set at 220 nm) of **1**. (B) UV spectrum of **1** in LC/MS analysis. (C) Negative ion-mode ESI MS data of **1** in LC/MS analysis.

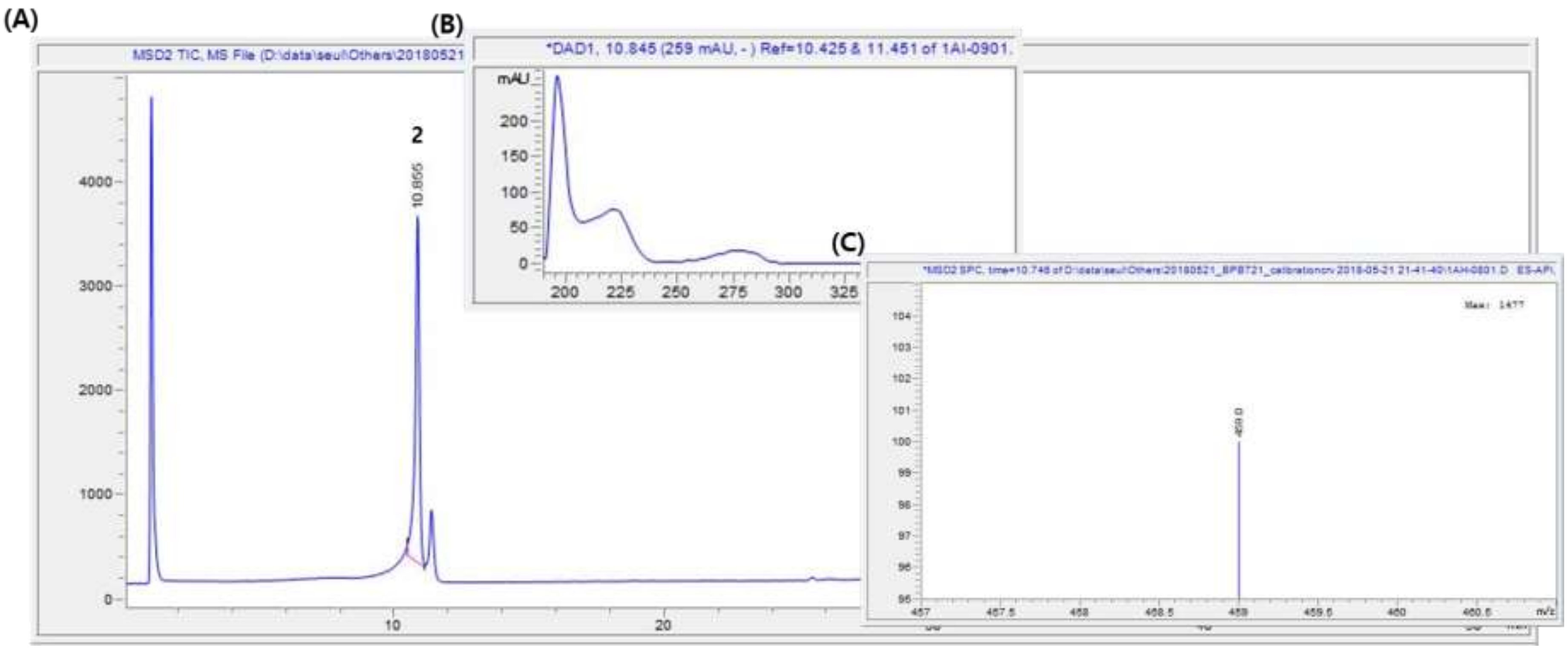

Figure S2. The LC/MS analysis of compound **2**. (A) UV chromatogram of LC/MS (detection wavelength was set at 220 nm) of **2**. (B) UV spectrum of **2** in LC/MS analysis. (C) Negative ion-mode ESI MS data of **2** in LC/MS analysis.

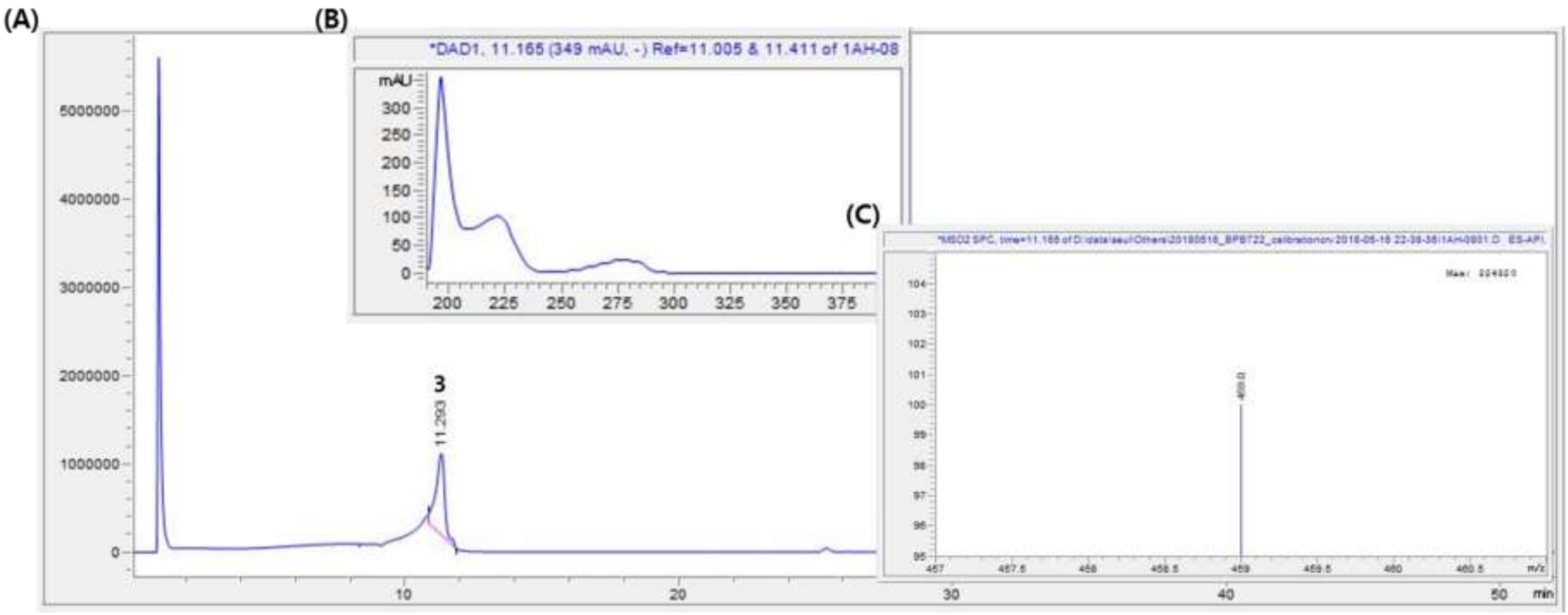

Figure S3. The LC/MS analysis of compound **3**. (A) UV chromatogram of LC/MS (detection wavelength was set at 220 nm) of **3**. (B) UV spectrum of **3** in LC/MS analysis. (C) Negative ion-mode ESI MS data of **3** in LC/MS analysis.

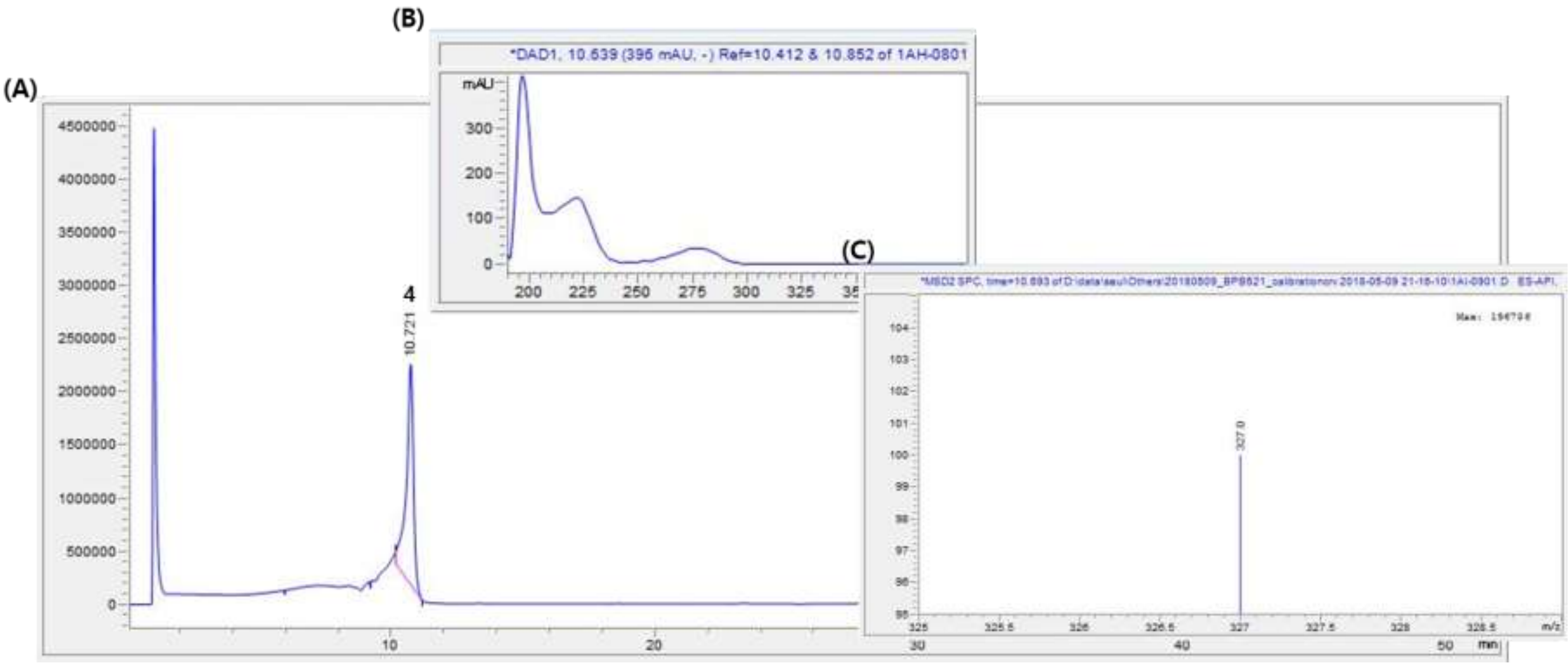

Figure S4. The LC/MS analysis of compound **4**. (A) UV chromatogram of LC/MS (detection wavelength was set at 220 nm) of **4**. (B) UV spectrum of **4** in LC/MS analysis. (C) Negative ion-mode ESI MS data of **4** in LC/MS analysis.

LC/MS analysis of the EtOH extract and the *n*-BuOH-soluble fraction

The EtOH extract of *B. platyphylla* var. *japonica* bark and the *n*-BuOH-soluble fraction were analyzed by LC/MS. Stock solutions of the EtOH extract and the fraction were prepared by dissolving 1 mg of sample in 1 mL methanol, to provide a solution of 1000 μg/mL. The solutions were filtered through a 0.45 mm hydrophobic PTFE filter and analyzed by LC/MS (Agilent Technologies, Santa Clara, CA, USA) using a LC-MS Agilent 1200 Series analytical system equipped with a photodiode array (PDA) detector combined with a 6130 Series ESI mass spectrometer. Analysis was performed by the injection of 15 μL of each sample using a Kinetex C18 column (2.1 × 100 mm, 5 μm; Phenomenex, Torrance, CA, USA) set at 25°C. The mobile phase consisting of formic acid in H_2O [0.1% (v/v)] (A) and methanol (B) was delivered at a flow rate of 0.3 mL/min by applying the following programmed gradient elution: 10%-90% (B) for 30 min, 100% (B) for 1 min, 100% (B) isocratic for 10 min, and then 10% (B) isocratic for 10 min, to perform post-run reconditioning of the column.

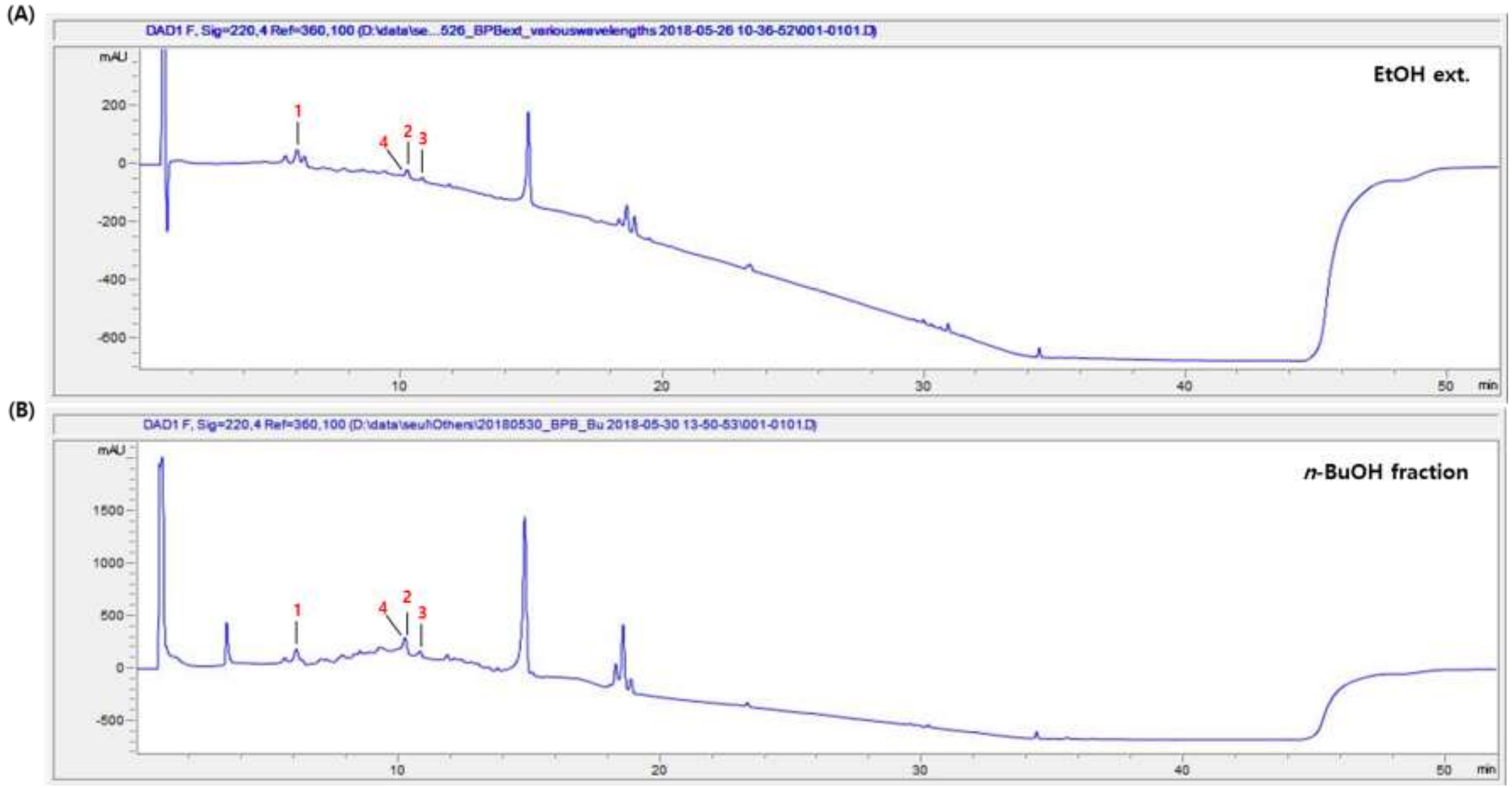

Figure S5. The LC/MS analysis of the EtOH extract and the *n*-BuOH soluble fraction for compounds **1**-**4**. UV chromatogram of LC/MS (detection wavelength was set at 220 nm) of (A) the EtOH extract, and (B) the *n*-BuOH soluble fraction.
